# A Dual-Branch Spatial Interaction and Multi-Scale Separable Aggregation Driven Hybrid Network for Infrared Image Super-Resolution

**DOI:** 10.3390/s26041332

**Published:** 2026-02-19

**Authors:** Jiajia Liu, Wenxiang Dong, Xuan Zhao, Jianhua Liu, Xiaoguang Tu

**Affiliations:** 1 Faculty Development and Teaching Evaluation Center, Civil Aviation Flight University of China, Guanghan 618307, China; 2 College of Aviation Electronic and Electrical Engineering, Civil Aviation Flight University of China, Guanghan 618307, China; xiangwendong2002@163.com (W.D.); zhaoxuangood@cafuc.edu.cn (X.Z.); jianhuacafuc13@cafuc.edu.cn (J.L.); xguangtu@cafuc.edu.cn (X.T.)

**Keywords:** attention mechanism, convolutional neural network, infrared image super-resolution, spatial interaction, multi-scale feature aggregation

## Abstract

Single image super-resolution (SISR) is a classical computer vision task that aims to reconstruct a high-resolution image from a low-resolution input, thereby improving detail sharpness and visual quality. In recent years, convolutional neural network (CNN)-based methods and transformer-based methods using self-attention mechanisms have achieved significant progress in visible-image super-resolution. However, the direct application of these two types of methods to infrared images still poses considerable challenges. On the one hand, infrared images generally suffer from low signal-to-noise ratio, blurred edges, and missing details, and relying only on local convolutions makes it difficult to adequately model long-range dependencies across regions. On the other hand, although pure transformer models have a strong global modeling ability, they usually have large numbers of parameters and are sensitive to the amount of training data, making it difficult to balance efficiency and detail restoration in infrared imaging scenarios. To address these issues, we propose a hybrid neural network architecture for infrared image super-resolution reconstruction, termed RDSR (Residual Dual-branch Separable Super-Resolution Network), which organically integrates multi-scale depthwise separable convolutions with shifted-window self-attention. Specifically, we design a dual-branch spatial interaction module (BDSI, Dual-Branch Spatial Interaction) and a multi-scale separable spatial aggregation module (MSSA, Multi-Scale Separable Spatial Aggregation). The BDSI module models correlations along rows and columns through grouped convolutions in the horizontal and vertical directions, effectively strengthening the spatial information interaction between the convolution branch and the self-attention branch. The MSSA module replaces the conventional MLP with three parallel depthwise separable convolution branches, improving the feature representation and nonlinear modeling through multi-scale spatial aggregation and a star-shaped gating operation. The experimental results on multiple public infrared image datasets show that for ×2 and ×4 upscaling, the proposed RDSR achieves higher PSNR and SSIM values than CNN-based methods such as EDSR, RCAN, and RDN, as well as transformer-based methods such as SwinIR, DAT, and HAT, demonstrating the effectiveness of the proposed modules and the overall framework.

## 1. Introduction

Infrared imaging, with the advantages of passive thermal radiation sensing and robustness to illumination and weather conditions, has become an indispensable perception modality in fields such as nighttime security surveillance, aerospace remote sensing, and medical thermal diagnosis. However, due to the hardware limitations of infrared cameras (e.g., low detector resolution) and imaging characteristics (e.g., weak temperature contrast and scarce texture details), infrared images commonly suffer from insufficient spatial resolution, smooth edge gradients, and blurred target details. These issues directly limit the accuracy of downstream computer vision tasks such as object detection and semantic segmentation. Therefore, infrared image super-resolution (IRSR) aims to reconstruct high-resolution images from low-resolution infrared inputs and has become a key approach for improving the practical value of infrared imagery [1].

Single-image super-resolution (SISR) aims to reconstruct high-resolution images from low-resolution inputs, and its development has been driven by two mainstream deep learning frameworks: convolutional neural networks (CNNs) and transformers with self-attention mechanisms [2]. CNN-based SISR methods rely on local inductive bias to achieve excellent fine-grained texture and edge recovery, and representative methods such as SRCNN [3] and VDSR [4] laid the foundation for deep learning-based super-resolution; the EDSR [5], RDN [6] and RCAN [7] models involved in this study further enhanced local feature representation through residual dense connections and channel attention mechanisms, yet still failed to break through the limitation of local modeling and cannot efficiently capture long-range spatial dependencies. Transformer-based SISR methods address the above shortcomings by explicitly computing the correlation between arbitrary spatial positions, with SwinIR [8] pioneering window-based hierarchical self-attention for efficient global modeling; DAT [9], HAT [10] and other improved models optimize the attention calculation logic to enhance the global dependency modeling ability, but they usually suffer from large parameter counts and high computational costs, and show poor generalization and overfitting risks in infrared imaging scenarios with limited training data and complex noise. Thus, CNN-transformer hybrid architectures have become the mainstream research direction for SISR, which combine the local detail modeling advantage of CNNs and the global dependency modeling advantage of transformers to balance performance and efficiency.

For IRSR, existing hybrid models like InfraFFN [11] have pioneered this fusion idea. However, they are not fully tailored to the unique characteristics of infrared imagery—namely, large uniform thermal regions coupled with sparse key edges. We identify two critical limitations in current hybrid frameworks. First, the interaction between CNN and self-attention branches is often superficial, lacking an explicit mechanism to fuse local features with global dependencies. This results in poor structural consistency within large thermal regions. Second, the standard feed-forward network (FFN) typically employs a simple multilayer perceptron (MLP), which lacks multi-scale spatial aggregation abilities. This limits the network’s ability to capture the sparse textures and subtle edge gradients characteristic of infrared scenes.

To address the above limitations of existing IRSR hybrid architectures (especially InfraFFN [11]) and fully exploit the synergy between CNN and transformer for infrared image characteristics, this paper proposes a Residual Dual-branch Separable Super-Resolution Network (RDSR) for IRSR, which organically combines multi-scale depthwise separable convolutions with shifted-window self-attention. The core innovation of RDSR lies in designing two novel modules to optimize the hybrid architecture for infrared imaging characteristics: (1) A Dual-Branch Spatial Interaction (BDSI) module, which models correlations along rows and columns through horizontal and vertical grouped convolutions, effectively strengthening the spatial information interaction between the convolution branch and the self-attention branch; (2) A Multi-Scale Separable Spatial Aggregation (MSSA) module, which replaces the conventional MLP with three parallel depthwise separable convolution branches, improving feature representation and nonlinear modeling through multi-scale spatial aggregation and a star-shaped gating operation. Based on a residual dense backbone, RDSR integrates the BDSI and MSSA modules to realize the deep fusion of local convolutional features and global attention features, thus better adapting to the structural characteristics of infrared images.

The main contributions of this paper are summarized as follows:

- We propose a residual dual-branch convolution-self-attention interaction network (RDSR, Residual Dual-branch Separable Super-Resolution Network) for IRSR, which is designed to improve the performance of InfraFFN by addressing its core limitations in branch interaction and spatial aggregation. The network adopts a residual dense backbone to build parallel CNN and self-attention branches, laying a foundation for the deep fusion of local and global features.
- We design the BDSI module based on horizontal and vertical grouped convolutions and dual-gated fusion. This module explicitly models the intra-row and inter-column spatial correlations of infrared images, enhances the bidirectional spatial feature interaction between CNN and self-attention branches, and effectively improves the structural consistency of long edges and large uniform thermal regions in reconstructed infrared images.
- We propose the MSSA module as a lightweight alternative to the traditional MLP in FFN. MSSA adopts three parallel depthwise separable convolution branches with different kernel sizes to realize multi-scale spatial feature aggregation, which effectively captures the sparse texture and weak edge features of infrared images while reducing the computational cost of the network.
- Extensive experimental results on multiple public infrared super-resolution datasets demonstrate that the proposed RDSR with BDSI and MSSA significantly outperforms state-of-the-art CNN-based, transformer-based, and hybrid methods in both quantitative metrics (PSNR/SSIM) and visual quality. The ablation experiments further verify the effectiveness and necessity of the BDSI and MSSA modules for improving the performance of IRSR.

The remainder of this paper is organized as follows: Section 2 reviews the related work on infrared image super-resolution, covering traditional methods and various deep learning architectures. Section 3 elaborates on the network architecture of RDSR and the design principles of its core modules (BDSI and MSSA). Section 4 presents experimental results, including dataset descriptions, comparative experiments, model complexity analysis, and ablation studies. Finally, Section 5 concludes the paper and discusses future research directions.

## 2. Related Work

As a key technique for overcoming hardware limitations and improving image spatial resolution through computational methods, image super-resolution has evolved from traditional approaches to deep learning-based methods. In infrared thermography, the PBVS Thermal Image Super-Resolution challenge has provided unified datasets and evaluation protocols in recent years, promoting the fair comparison and rapid iteration of methods [12].

### 2.1. Traditional Super-Resolution Methods

Traditional super-resolution methods can be mainly divided into interpolation-based, reconstruction-based, and frequency-domain approaches. Park et al. introduced interpolation-based super-resolution methods, which upscale low-resolution images via spatial interpolation [13]. These methods are computationally efficient and easy to implement, meeting the need for fast upscaling under limited hardware conditions. However, they do not explicitly model the degradation and noise processes, and thus cannot reliably recover high-frequency details; at high upscaling factors and in complex-texture scenes, they often produce obvious blur and jagged artifacts. To alleviate the over-smoothing of interpolation methods, Farsiu et al. proposed reconstruction-based super-resolution methods that introduce sparse or prior regularization under the constraints of an imaging degradation model and estimate high-resolution images by minimizing reconstruction error [14]. Such methods can improve noise suppression and detail recovery when multi-frame observations are available or prior information is accurate. However, their performance strongly depends on the degradation model and prior assumptions; once they deviate from the real imaging process, the reconstruction quality degrades significantly, and iterative optimization introduces considerable computational costs. To better recover details through multi-resolution analysis, Temizel and Vlachos [15] proposed a wavelet-domain enhancement method: the image is first decomposed by wavelet transform into low- and high-frequency subbands at different scales, the high-frequency detail subbands are then estimated and enhanced, and finally the high-resolution image is reconstructed by inverse wavelet transform, thereby improving high-frequency information such as edges and textures. These traditional methods are theoretically clear, interpretable, and computationally inexpensive, but they often struggle to recover high-frequency details under large magnification, complex degradation, and low-texture conditions.

### 2.2. CNN-Based Super-Resolution Methods

The core limitation of traditional methods is their limited representational capacity, precluding the automatic learning of complex features. Therefore, researchers began to use CNNs to directly learn the mapping from low resolution to high resolution from data. In 2015, Dong et al. proposed SRCNN [3], which first introduced a shallow CNN into super-resolution and achieved better results than traditional methods, opening the era of deep learning for this task. However, due to its limited network depth, SRCNN is still insufficient for recovering complex textures and handling large upscaling factors. To improve representational ability, Kim et al. proposed VDSR [4], building a deep convolutional network with more than twenty layers and introducing residual learning to significantly improve reconstruction accuracy. Kim et al. further proposed DRCN [16], which uses a recursive convolutional structure to share parameters, expanding effective depth while controlling parameter size. These works indicate that increasing depth and using residual/recursive structures can improve super-resolution performance, but they also lead to larger models and more difficult training, and they still rely on local convolutions with limited ability to explicitly model long-range structural relationships. Afterwards, SRResNet [17] and EDSR [5] systematically introduced residual learning into super-resolution networks; by removing batch normalization and increasing channel width, these networks became more suitable for high-precision image restoration. RDN [6] and RRDB [18] further combine residual and dense connections to strengthen feature transmission and reuse across layers, achieving better performance on complex textures and structural detail recovery. As network designs matured, RCAN [7] and HAN [19] introduced channel attention, spatial attention, and layer attention into CNN frameworks to explicitly model the importance of different channels and spatial locations, enhancing high-frequency detail and structural reconstruction. These CNN-based methods have achieved significant success in visible-image super-resolution and largely address the limited representational capacity of traditional approaches. However, because they are based on convolution kernels of limited size, even with deep stacking or dilated convolutions, their modeling range is still mainly restricted to local receptive fields and they cannot fully capture long-range dependencies. In infrared images with complex noise, low contrast, and sparse textures, this limitation becomes more prominent and can easily lead to over-smoothed details and loss of structural information.

### 2.3. Transformer-Based Super-Resolution Methods

CNN-based methods rely on local kernels and thus have difficulty explicitly modeling long-range dependencies, even with deep networks or dilated convolutions. To address this issue, self-attention and transformer architectures have been introduced into super-resolution. ViT proposed by Dosovitskiy et al. [20] demonstrated the feasibility of standard transformers for image classification, and self-attention has since been widely applied to detection, segmentation, and image restoration tasks. Unlike local convolutions, transformers explicitly compute correlations between arbitrary positions in the feature sequence through self-attention, enabling long-range dependency modeling and non-local structure reasoning. This effectively mitigates CNNs’ insensitivity to long-distance structures and improves the recovery of large-scale textures and global structures. However, the computational complexity of global self-attention grows quadratically with feature resolution, significantly increasing memory usage and inference time for high-resolution reconstruction. To reduce this cost, Liang et al. proposed SwinIR [8], which partitions features into non-overlapping windows and computes self-attention only within each window based on the hierarchical structure of Swin Transformer. This reduces computation while retaining a certain degree of global modeling capability, achieving strong performance in denoising, deraining, and visible-image super-resolution. For infrared images, DASR [21] introduces dual-attention transformers to model feature dependencies in both spatial and channel dimensions, thereby enhancing long-range context capture. In addition, Kansal et al. [22] proposed a dual-input frequency-aware network that decomposes the input into different frequency bands and selectively enhances and fuses key bands, providing a more targeted way to handle infrared imaging characteristics such as low contrast, blurred edges, and sparse details. Nevertheless, transformer-based methods also face challenges. First, self-attention has quadratic complexity with respect to feature resolution, leading to large parameter counts and high computational cost, and requiring substantial training data and hardware resources. Second, window-based self-attention such as SwinIR often computes attention only within fixed-size local windows, making it difficult to fully exploit long-range dependencies beyond the window boundaries. Moreover, many architectures are derived from high-level vision tasks and lack a dedicated modeling of degradation mechanisms and noise distributions for super-resolution, especially in infrared scenarios. Therefore, relying solely on transformers makes it difficult to simultaneously achieve strong global modeling, fine local detail rendering, and high efficiency in infrared super-resolution.

### 2.4. CNN–Transformer Hybrid Super-Resolution Methods

As discussed above, CNNs are effective at modeling local neighborhood structures, while self-attention is more suitable for modeling long-range dependencies. Their complementary properties have motivated hybrid architectures that exploit both types of modules. In speech recognition, Gulati et al. proposed Conformer [23], embedding convolution modules into self-attention encoders to jointly model local and global dependencies in a parameter-efficient manner, and achieving better performance than models using only transformers or only CNNs. In medical image segmentation, Guo et al. [24] proposed parallel heterogeneous modules that combine convolution and self-attention within the same block and achieved significant improvements on multiple segmentation datasets. In image classification, object detection, and instance segmentation, Srinivas et al. [25] replaced part of the spatial convolutions in ResNet with global self-attention and improved performance without changing other components. For low-level vision tasks, Quan et al. [26] proposed a complementary cascaded network that cascades CNN blocks and self-attention blocks and combines neural architecture search, achieving superior results in image deraining. Pan et al. [27] analyzed the commonality of convolution and self-attention at the operator level, unifying them as special forms of 1×1 convolution projection followed by weighted aggregation, and designed hybrid modules where convolution and attention branches process features in parallel and are fused at the feature level.

For infrared image super-resolution, several representative hybrid models emerged in 2025. For example, InfraFFN [11] models local convolution and global self-attention in two branches and enhances infrared detail recovery through feature fusion blocks. MIHNet [28] adopts a multi-input hierarchical encoder–decoder framework to coordinate CNNs and transformers. PIFRNet [29] introduces hybrid CNN–transformer modules in cascaded feature refinement paths and constructs a dedicated infrared SR dataset. For lightweight designs, CDSMANet [30] enables local–non-local feature interaction and efficiency through contrast-driven self-modulated aggregation. These studies indicate that convolution–self-attention hybrid structures can effectively alleviate the modeling limitations of single-structure networks in tasks such as speech, medical image analysis, image restoration, and reconstruction. In addition, relevant research in infrared image enhancement also provides important support for IRSR. U2D2Net [31] proposes an unsupervised unified framework for image dehazing and denoising, which is beneficial for preprocessing low-quality infrared inputs and suppressing complex noise that affects super-resolution performance. Meanwhile, the detail-aware network [32] is specially designed to enhance fine structures and weak edges in infrared images, which provides valuable insights for detail preservation in infrared super-resolution reconstruction.

However, most existing hybrid models are designed for speech or visible natural images, and tailored research for IRSR remains limited. On the one hand, many hybrid networks adopt serial or simple parallel strategies, and the interaction between convolution and attention branches mainly remains at the block or stage level, lacking fine-grained spatial interaction modeling [33]. On the other hand, these networks are designed based on the statistical characteristics of visible images and do not sufficiently consider the structural properties of infrared images—large uniform regions plus a few key edges—as well as complex noise and non-uniform response. When directly transferred to infrared super-resolution, it becomes difficult to achieve an ideal balance between performance gains and additional computational cost, and there is still room to improve both long-range structural reconstruction and local detail depiction. To address the aforementioned issues, we design the BDSI module based on horizontal/vertical grouped convolutions to enhance row/column correlation modeling and inter-branch information flow, thereby effectively improving structural consistency in both edge details and thermal-region distributions. At the same time, to address the limited spatial utilization of the MLP in the forward propagation of the FFB and its relatively high parameter and computational overhead, we replace the MLP with the MSSA module based on multi-scale depthwise separable convolution spatial aggregation. This enables more lightweight multi-scale spatial feature fusion and nonlinear enhancement, thereby improving detail restoration and global consistency for infrared super-resolution under controllable efficiency.

## 3. Method Principles

### 3.1. Network Architecture

**Figure 1:**
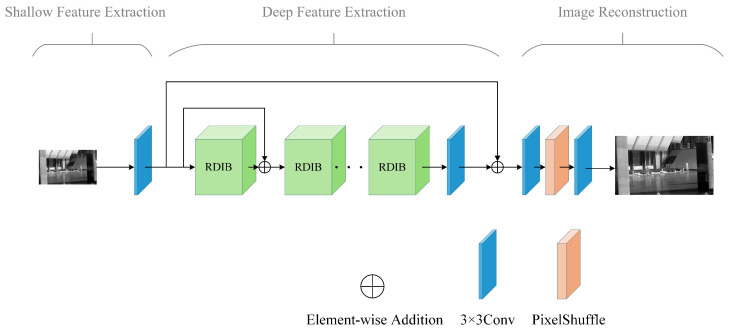
The proposed infrared image super-resolution network, termed RDSR.

This section presents our proposed architecture (as shown in Figure 1), which consists of three modules: a shallow feature extraction module, a deep feature extraction module, and an image reconstruction module. This design is tailored for infrared images, which are characterized by weak details and crucial edge information. The proposed network model, referred to as RDSR, is shown in Figure 1.

(1) Shallow Feature Extraction Module: Given an input low-resolution (LR) infrared image $I_{LR}\in R^{H\times W\times C}$, where *H*, *W*, and *C* denote the height, width, and number of channels of the image, respectively, a 3×3 convolutional layer is used to extract basic texture and edge responses, yielding shallow representations:

(1) $$F_{0}=S\left(I_{LR}\right)={\mathrm{Conv}}_{3\times 3}\left(I_{LR}\right),F_{0}\in R^{H\times W\times C}$$

where *C* represents the number of channels, and ${\mathrm{Conv}}_{3\times 3}\left(\cdot \right)$ denotes a 3×3 convolution operation [3]. This stage primarily maps the input from low-dimensional space to high-dimensional space, extracting low-frequency information from the image.

(2) Deep Feature Extraction Module: The shallow features $F_{0}$ are input into the deep feature extraction module to expand the receptive field and improve the ability to characterize structural details. The deep feature extraction module is the core component of the network, consisting of multiple stacked Residual Dual-branch Interaction Blocks (RDIBs) and a 3×3 convolution layer, and finally extracts deep features $F_{d}\in R^{H\times W\times C}$. The process is as follows:

(2) $$F_{M}=RDIB_{M}\left(\cdot \cdot \cdot \left(RDIB_{1}\left(F_{0}\right)\right)+F_{0}\right)+F_{M-1},\;M=1,2,\ldots ,N$$

(3) $$F_{d}={\mathrm{Conv}}_{3\times 3}\left(F_{M}\right)+F_{0}$$

where *N* is the number of residual feature fusion blocks, and $RDIB_{i}\left(\cdot \right)$ denotes the *i*-th RDIB block. For the input feature $F_{i-1}$ of the *i*-th RDIB block, feature extraction is first performed using the RDIB, and then a residual connection is made with the input features. This can be expressed as follows:

(4) $$F_{i}=RDIB_{i}\left(F_{i-1}\right)+F_{i-1}$$

(3) Image Reconstruction Module: To enhance gradient propagation and improve training stability, a skip connection is used to merge deep features with shallow features. The merged result is then passed into the reconstruction module to generate the final super-resolved image $F_{out}$:

(5) $$F_{out}={\mathrm{Conv}}_{3\times 3}\left(\mathrm{PixelShuffle}\left({\mathrm{Conv}}_{3\times 3}\left(F_{d}\right)\right)\right)$$

The PixelShuffle operation can achieve high-quality upsampling with a small computational cost, avoiding over-smoothing caused by interpolation [3]. The model uses the $L_{1}$ loss between the reconstructed image ($F_{out}$) and the corresponding high-resolution image ($I_{HR}$) for optimization:

(6) $$L_{1}=\arg\min(∥F_{out}-I_{HR}∥)$$

### 3.2. Residual Dual-Branch Interaction Block (RDIB)

**Figure 2:**
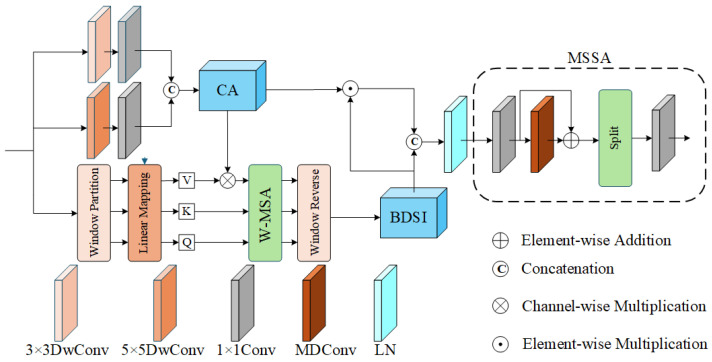
The architecture of the RDIB module.

Infrared images typically suffer from inherent degradation factors, such as low thermal contrast, sparse texture information, and blurred structural edges, which stem from the physical limitations of infrared sensors. In the context of super-resolution reconstruction, relying solely on local convolution often fails to recover long-range structural dependencies, while a single global attention mechanism may struggle to preserve fine-grained edge details. To address this imbalance between detail enhancement and structural consistency, this paper proposes a Residual Dual-branch Interaction Block (RDIB), as illustrated in Figure 2.

The core design philosophy of RDIB lies in the parallel processing of multi-scale features. Specifically, the input features are simultaneously projected into two specialized paths: a convolution branch and a self-attention branch. To facilitate feature synergy, Channel Attention (CA) and Multi-Scale Self-Attention (MSSA) modules are integrated between these branches for bidirectional information complementation. This architectural choice is inspired by the dual-branch interaction design of the InfraFFN model [11], where the CA module serves as a traditional mechanism to recalibrate channel-wise importance [34]. By executing feature extraction in parallel, the model can effectively capture local spatial correlations while maintaining a global receptive field. The detailed mechanisms of these two branches and their interaction logic will be elaborated in the following sections.

**Convolution Branch:** The effective information of infrared images is often concentrated at edges, contours, and local gradient mutations, and the target scale varies greatly. Therefore, using a single convolution kernel is prone to problems such as insufficient receptive field or over-smoothing. To this end, this model introduces parallel multi-scale local convolution paths within the block. This branch expands the effective receptive field through the parallel depthwise separable convolution kernels of different sizes, and combines channel remapping to achieve the fine-grained modeling of local structures. The specific process is as follows: Let the input feature of RDIB be $F\in R^{H\times W\times C}$. First, it passes through 3×3 and 5×5 depthwise separable convolution branches [35], and the results are calculated as follows:

(7) $$F_{1}={\mathrm{DwConv}}_{3\times 3}\left(F\right),\;F_{2}={\mathrm{DwConv}}_{5\times 5}\left(F\right)$$

where ${\mathrm{DwConv}}_{k\times k}\left(\cdot \right)$ denotes the depthwise separable convolution operation with a kernel size of k×k. Depthwise separable convolution performs spatial convolution independently on each channel, only focusing on local patterns in the spatial neighborhood, which significantly reduces the number of parameters and computational complexity compared with standard convolutions of the same kernel size. Intuitively, the 3×3 branch is more sensitive to small-scale texture and edge information, while the 5×5 branch focuses more on depicting target contours and relatively smooth thermal distributions. Subsequently, both branches are subjected to 1×1 convolution for channel dimensionality reduction, and then the outputs of the two reduced-dimensional branches are concatenated in the channel dimension to obtain multi-scale responses, which are specifically expressed as follows:

(8) $$F_{1}^{\prime }={\mathrm{Conv}}_{1\times 1}\left(F_{1}\right),\;F_{2}^{\prime }={\mathrm{Conv}}_{1\times 1}\left(F_{2}\right),\;F_{m}=\mathrm{Concat}\left(F_{1}^{\prime },F_{2}^{\prime }\right)\in R^{H\times W\times C}$$

**Self-Attention Branch:** To capture global information, we introduce an attention mechanism for global feature modeling. Self-attention generates corresponding query (*Q*), key (*K*), and value (*V*) vectors for each element in the input sequence; then, the attention score is obtained by calculating the dot product of each query vector with all key vectors and scaling, and the score is normalized to weights through the Softmax function. Finally, the weights are multiplied by the corresponding value vectors and summed to output a new vector representation integrating global context information for each position. Its core formula can be concisely expressed as follows:

(9) $$\mathrm{Attention}\left(Q,K,V\right)=\mathrm{Softmax}\left(\frac{QK^{T}}{\sqrt{d_{k}}}\right)V+B$$

where *d* is the embedding dimension, and *B* is the learnable relative position bias [20]. Although self-attention can effectively model long-range information and capture global features, directly applying it to super-resolution tasks will over-weight noise information and lead to huge computational costs.

Therefore, this study draws on the window attention mechanism of SwinIR to limit global attention within local windows to reduce computational costs and improve robustness [8]. Specifically, given spatial features $X\in R^{H\times W\times C}$, the features are first divided into several windows of size M×M through Window Partition and we rearranged it into a window sequence representation $X_{w}\in R^{nW\times M^{2}\times C}$ (where *n* is the number of windows). Subsequently, self-attention is calculated within each window, thereby modeling the local-context relationships within the window under controllable complexity. Finally, the window sequence is restored to the spatial feature form through Window Reverse to obtain the output of the attention branch $F_{attn}\in R^{H\times W\times C}$. Through windowed processing, the computational complexity of global attention is reduced from $O({\left(HW\right)}^{2})$ to $O(HW\cdot M^{2})$, which is more suitable for high-resolution feature recovery tasks such as super-resolution. At the same time, intra-window attention can strengthen local structural consistency while suppressing noise interference, and provide a more robust global structural prior for subsequent interaction with the convolution branch. To further realize information flow across windows, this study adopts a shifted window strategy in adjacent layers, enabling indirect connections between different windows, thereby balancing local detail modeling and larger-scale structural consistency. It should be noted that the main structure of the attention branch in this paper follows the window attention paradigm of InfraFFN, and the main improvements are concentrated on the inter-branch spatial interaction module BDSI and the feed-forward aggregation module MSSA. The following two modules will be introduced.

### 3.3. Dual-Branch Spatial Interaction (BDSI) Module

**Figure 3:**
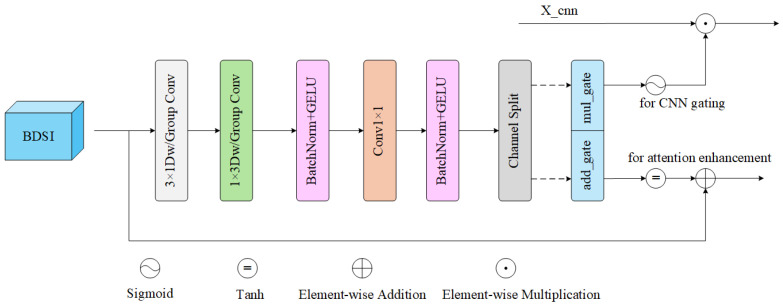
The architecture of the BDSI module.

The key to the dual-branch structure lies in how to achieve effective information exchange. If only simple fusion is performed at the block level or stage level, this can easily lead to insufficient complementarity between local details and global structures. To this end, this paper proposes the BDSI module, as shown in Figure 3.

This module uses horizontal and vertical grouped convolutions to explicitly model row-column correlations, promoting fine-grained interaction between the convolution branch and the attention branch in the spatial dimension, thereby making them more suitable for the structural distribution of large-area smooth regions and a small number of key edges in infrared images.

Given the convolution branch output $F_{m}\in R^{H\times W\times C}$ and the self-attention branch output $F_{attn}\in R^{H\times W\times C}$, the module is driven by the spatial features of the attention branch and adopts two-step decomposed directional grouped convolutions to extract spatial dependencies:

(10) $$U={\mathrm{GConv}}_{3\times 1}\left(F_{attn}^{sp}\right)\to {\mathrm{GConv}}_{1\times 3}\left(U\right)$$

where ${\mathrm{GConv}}_{3\times 1}$ and ${\mathrm{GConv}}_{1\times 3}$ correspond to grouped convolutions with kernel sizes of (3,1) and (1,3) respectively. Through the decomposition method of first vertical then horizontal, the directional continuity of long edges, strip-shaped structures, and large-area thermal regions commonly found in infrared images can be captured with low computational cost. After convolution, BatchNorm and GELU activation are connected:

(11) U=GELU(BN(U))

After obtaining the directional interaction features *U*, two spatial gating maps are generated through 1×1 convolution and split along the channel to obtain the following:

(12) $$G_{mul},G_{add}=\mathrm{chunk}\left(U,2\right)\in R^{B\times C\times H\times W}$$

where *B* denotes the batch size, *C* the number of channels, and H,W the spatial height and width. Subsequently, BDSI performs different forms of modulation on the dual branches. Multiplicative modulation is performed on the convolution branch:

(13) $$X_{cnn}^{\prime }=\sigma \left(G_{mul}\right)⊙X_{cnn}$$

where σ(·) is the Sigmoid activation function, and ⊙ is the element-wise multiplication. The output of Sigmoid is in [0,1], which is suitable for spatial position gating, suppressing background noise and highlighting key edge regions. Additive enhancement is performed on the attention branch:

(14) $$X_{attn}^{\prime }=X_{attn}^{sp}+\tanh\left(G_{add}\right)$$

where the tanh(·) output is in [−1,1], which performs bounded enhancement on the attention features in the form of residuals, avoiding unstable attention branches caused by excessive disturbance. Finally, the updated $X_{attn}^{\prime }$ is re-partitioned into token form through windows for subsequent attention calculation.

BDSI generates dual gating maps based on the spatial representation of the attention branch, performing multiplicative filtering on the CNN branch and additive enhancement on the attention branch respectively, using guidance from global structures for local details and compensation from local information for global representations, thereby improving the structural consistency and detail performance of infrared image super-resolution reconstruction. Such a reasonable spatial interaction mechanism is one of the key factors for the superior performance of the proposed model.

### 3.4. Multi-Scale Separable Spatial Aggregation (MSSA) Module

**Figure 4:**
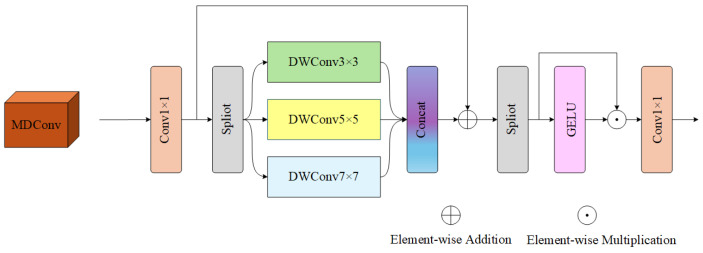
The architecture of the MDConv module.

The feed-forward network (FFN) in traditional Transformer hybrid blocks is typically realized with a vanilla Multi-Layer Perceptron (MLP), which only performs simple channel-wise mixing on flattened sequence features with an indirect spatial aggregation ability, a single nonlinear modulation mode, and relatively high parameter overhead. To address these inherent drawbacks of MLP and adapt to the feature distribution of infrared images (e.g., sparse textures, weak edges, and block-wise continuous thermal regions), this paper proposes the Multi-Scale Separable Spatial Aggregation (MSSA) module to replace the traditional MLP as an integral FFN component within the Residual Dual-branch Interaction Block (RDIB). The MSSA module is composed of 1 × 1 point convolutions for dimension scaling, a custom Multi-Scale Depthwise Convolution (MDConv) submodule for multi-scale spatial feature extraction, residual connections for feature enhancement, and star-shaped gating for adaptive nonlinear modulation. Specifically, the architecture of the MDConv submodule (the core spatial aggregation component of MSSA) is illustrated in Figure 4.

Taking the spatial-format feature adapted for convolutional operations as input, denoted as $X\in R^{B\times C\times H\times W}$, where *B* is the batch size, *C* is the channel dimension, and H,W are the spatial height and width of the feature map. First, a 1×1 convolution is used for channel upsampling to expand the feature representation space:

(15) $$Z={\mathrm{Conv}}_{1\times 1}\left(X\right),Z\in R^{B\times C_{h}\times H\times W}$$

where $C_{h}=\left\lfloor C\cdot r\right\rfloor$ and *r* is the MLPratio, consistent with the dimension expansion logic of the traditional MLP. Subsequently, *Z* is non-uniformly divided into three groups along the channel dimension (the first branch takes the residual channel to avoid feature loss caused by integer division, and the latter two are equally divided), and depthwise convolutions with different odd kernel sizes are applied to each group:

(16) $$Z=\left[Z^{\left(3\right)},Z^{\left(5\right)},Z^{\left(7\right)}\right],{\hat{Z}}^{\left(k\right)}={\mathrm{DWConv}}_{k\times k}\left(Z^{\left(k\right)}\right)$$

Here, DWConv denotes depthwise convolution with a group strategy, where the number of groups is set equal to the channel dimension of each group $Z^{\left(k\right)}$. This design enables independent spatial filtering for each channel while maintaining low parameter overhead. The outputs of the three paths are then concatenated along the channel dimension to restore the upsampled channel dimension:

(17) $$\hat{Z}=\mathrm{Concat}\left({\hat{Z}}^{\left(3\right)},{\hat{Z}}^{\left(5\right)},{\hat{Z}}^{\left(7\right)}\right)$$

To enhance multi-scale feature fusion and maintain the training stability of deep networks, residual stacking is adopted between the original upsampled feature and the multi-scale convolution feature:

(18) $$Z\leftarrow Z+\hat{Z}$$

Such a design can simultaneously capture small-scale edge details (3×3) and larger-scale thermal regions (5×5, 7×7) with low cost. After obtaining the enhanced *Z*, it is equally divided into two parts along the channel:

(19) $$Z_{1},Z_{2}=\mathrm{chunk}\left(Z,2\right)$$

And star-shaped operation is used to realize gating nonlinearity:

(20) $$S=\mathrm{GELU}\left(Z_{1}\right)⊙Z_{2}$$

That is, $Z_{1}$ is activated and then modulated with $Z_{2}$, thereby improving the representation ability and nonlinear expression at a low cost. Finally, the channel is projected back from $C_{h}$ to *C* through 1×1 convolution:

(21) $$Y={\mathrm{Conv}}_{1\times 1}\left(S\right),Y\in R^{B\times C\times H\times W}$$

As an integral improved alternative to the traditional MLP, the MSSA module is embedded into the forward propagation of the Transformer hybrid block via a lightweight sequence-to-spatial format conversion strategy. Based on the depthwise separable convolution, it explicitly performs multi-scale spatial aggregation while maintaining the lightweight property, which effectively makes up for the deficiency of MLP in spatial feature modeling. By replacing the traditional MLP and explicitly achieving multi-scale spatial aggregation, the MSSA module effectively improves the feature representation capability and nonlinear modeling ability. For infrared image super-resolution reconstruction, this module can significantly improve the detail recovery ability and structural consistency of the reconstructed images, which is the core reason for the high-quality resolution enhancement of the proposed method.

## 4. Experiments

This section verifies the effectiveness of the proposed model from both quantitative and qualitative perspectives. First, the datasets and evaluation indicators required for the experiments are introduced, and the relevant experimental details are elaborated; second, the experimental results of the model under different datasets for ×2 and ×4 magnification are compared with existing methods, and the comparison results are displayed; finally, the contributions of each proposed component are analyzed through ablation experiments.

### 4.1. Datasets and Evaluation Indicators

#### 4.1.1. Dataset Description

All training and test sets employed in the experiments are publicly available infrared image datasets. A brief description of the image types and acquisition methods for each dataset is provided as follows:

- **IR700 [36]** (Training Set): A long-wave infrared camera-captured dataset with various scenes, applicable for infrared image super-resolution. All images are cropped to 480 × 800 resolution.
- **IR700_test**: Test subset of the IR700 dataset, with the same image types and acquisition device as the training set.
- **Flir [37]**: Released by FLIR Systems Inc. (Wilsonville, OR, USA), captured by automotive-grade FLIR Boson 320 × 256 long-wave infrared cameras, including infrared images of urban/suburban road scenes, widely used for validating infrared image super-resolution and enhancement algorithms.
- **IR100 [38]**: Acquired by Guide infrared cameras, consisting of infrared images of outdoor targets (cars, drones, human bodies, etc.).
- **results-A [39]**: This dataset consists of 22 infrared images, and is commonly used for testing the performance of infrared image super-resolution (IRSR) models.
- **DLS-NUC-100 [40]**: Provided by the National University of Defense Technology, infrared images captured by cooled infrared detectors and processed with non-uniformity correction.

In summary, the selection criteria for these datasets were driven by the need to encompass a wide range of imaging conditions, including varying resolutions, sensor types (e.g., cooled vs. uncooled, industrial vs. consumer-grade), and diverse scenarios (e.g., industrial inspection vs. outdoor monitoring). Regarding the data partition, we adopted a cross-dataset evaluation protocol to rigorously test the model’s robustness. The IR700 dataset serves as the primary training source due to its high quality and representative thermal features. Conversely, the other five datasets (FLIR, IR100, results-A, DLS-NUC-100, and IR700_test) are strictly reserved as independent test sets. This design ensures that the evaluation is conducted on entirely unseen data from different sensors and environments, thereby providing a more convincing measure of the proposed method’s zero-shot generalization ability and practical reliability.

#### 4.1.2. Evaluation Metrics

Two evaluation indicators, Peak Signal-to-Noise Ratio (PSNR) and Structural Similarity Index (SSIM), were used for quantitative evaluation in the experiments. PSNR is a distortion metric defined based on the Mean Squared Error (MSE) between the reconstructed image and the reference high-resolution image, reflecting the proximity between the reconstructed result and the real image at the pixel intensity level. Its definition is as follows:

(22) $$PSNR=10\log_{10}\left(\frac{{\left(2^{n}-1\right)}^{2}}{MSE}\right)$$

(23) $$MSE=\frac{1}{M\cdot N}\sum_{i=0}^{M-1}\sum_{j=0}^{N-1}{\left[I\left(i,j\right)-K\left(i,j\right)\right]}^{2}$$

Among them, *n* is the number of bits per sample, *M* and *N* are the total number of pixels of images *I* and *K*, respectively, and MSE is the mean squared error between the reconstructed image and the reference image. The smaller the MSE value, the higher the image similarity. Therefore, the larger the PSNR value, the smaller the difference and distortion between the reconstructed image and the real image, and the higher the generation quality. SSIM measures the similarity between two images simultaneously from three aspects—brightness, contrast, and structure—and is closer to humans’ subjective perception. For the reference image *x* and the reconstructed image *y*, SSIM can be expressed as follows:

(24) $$SSIM\left(x,y\right)=\frac{\left(2\mu_{x}\mu_{y}+C_{1}\right)\left(2\sigma_{xy}+C_{2}\right)}{\left(\mu_{x}^{2}+\mu_{y}^{2}+C_{1}\right)\left(\sigma_{x}^{2}+\sigma_{y}^{2}+C_{2}\right)}$$

Among them, $\mu_{x}$ and $\mu_{y}$ are the brightness means of the images respectively, $\sigma_{x}^{2}$ and $\sigma_{y}^{2}$ are the variances, $\sigma_{xy}$ is the covariance, and $C_{1}$ and $C_{2}$ are stability constants. The value range of SSIM is usually in [0,1]; a larger value indicates that the two images are closer in structure and visual quality, so it is often used to evaluate the performance of super-resolution results in maintaining texture details and structural information.

### 4.2. Experimental Details

Experiments are conducted under two super-resolution scales: ×2 and ×4. The low-resolution images used in the experiments are generated by bicubic downsampling of the original high-resolution images. During training, the images are randomly cropped into 64×64 image patches, and the batch size is set to 2. The Adam optimizer is adopted, with parameters $\beta_{1}=0.9$ and $\beta_{2}=0.99$. The initial learning rate is set to $2\times 10^{-4}$, and the learning rate is halved when the number of iterations reaches 150 k, 200 k, 250 k, and 275 k, with a total of 300 k training iterations. The model is implemented based on the PyTorch 1.11.0 with CUDA 11.3 framework and trained and tested on an RTX 4090D graphics card.

### 4.3. Comparison with Existing Methods

#### 4.3.1. Quantitative Results

The RDSR model is compared with state-of-the-art CNN-based (e.g., EDSR [5], RCAN [7]) and Transformer-based methods (e.g., SwinIR [8], HAT [10], InfraFFN [11]). Table 1 summarizes the PSNR and SSIM metrics at ×2 and ×4 scales across five test sets.

The results show that RDSR achieves superior performance on all benchmarks. On IR700_test, RDSR outperforms EDSR by 0.30 dB and 0.66 dB at ×2 and ×4 scales, respectively. Compared with SwinIR, it yields PSNR gains of 0.07 dB (×2) and 0.32 dB (×4). Notably, the performance gap widens at ×4 scale, highlighting its robustness in recovering high-frequency details from degraded infrared images. RDSR also consistently ranks in the top two on Flir, IR100, and DLS-NUC-100 datasets. For instance, it reaches 36.66 dB at ×4 scale on DLS-NUC-100, 0.10 dB higher than InfraFFN. This strong generalization across scenarios stems from the dual-branch interaction, which synergistically fuses Transformer-based global dependencies with CNN-based local textures.

To verify the effectiveness of the proposed method in infrared image super-resolution reconstruction tasks, Table 2 provides the quantitative comparison results of the proposed method with representative infrared super-resolution models (such as DASR [21], CNNSC [36], etc.) under ×2 and ×4 magnification scales. The results of the proposed method are marked in bold in the table. It can be seen from Table 2 that in the ×4 magnification task, the proposed method RDSR achieves the best PSNR/SSIM on all test sets, surpassing other comparison methods and showing stronger detail recovery ability and structural fidelity. In the ×2 magnification task, the proposed method also maintains competitiveness, especially on datasets such as Flir and IR100, achieving a performance comparable to existing excellent methods. In summary, the proposed method exhibits excellent performance under different datasets and method scales, and has a good generalization ability.

In summary, the comprehensive experimental results across all evaluated datasets fully demonstrate the excellent generalization ability of the proposed RDSR model. Unlike most comparative general SR algorithms, which suffer from performance degradation on infrared-specific datasets, and dedicated IRSR models that only perform well on single or limited types of infrared datasets, our model maintains consistent and superior performance on diverse infrared datasets (including IR700, IR100, results-A, DLS-NUC-100, and FLIR ADAS) with different acquisition devices (industrial-grade FLIR cameras, Guide infrared cameras, cooled infrared detectors, etc.), scene types (industrial equipment inspection, outdoor target monitoring, road scenes, etc.), and resolution specifications. This outstanding cross-dataset performance verifies that the proposed model can effectively adapt to the inherent characteristics of various infrared images and overcome the limitations of dataset-specific overfitting, which fully confirms its strong generalization ability and practical application value for infrared image super-resolution tasks in real-world scenarios.

#### 4.3.2. Qualitative Results

**Figure 5:**
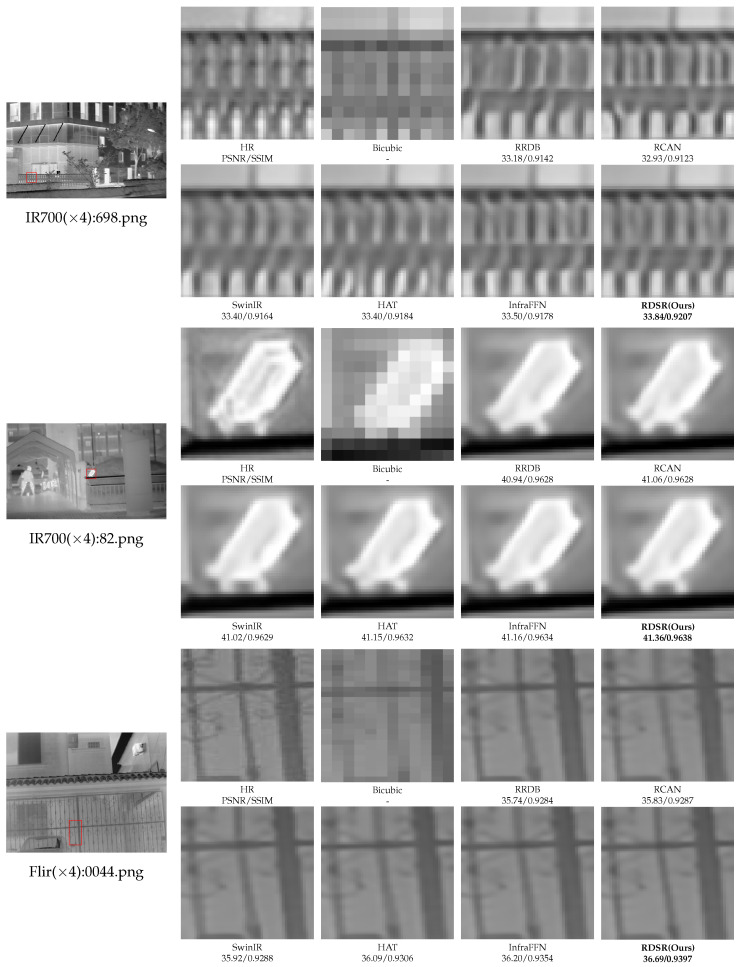

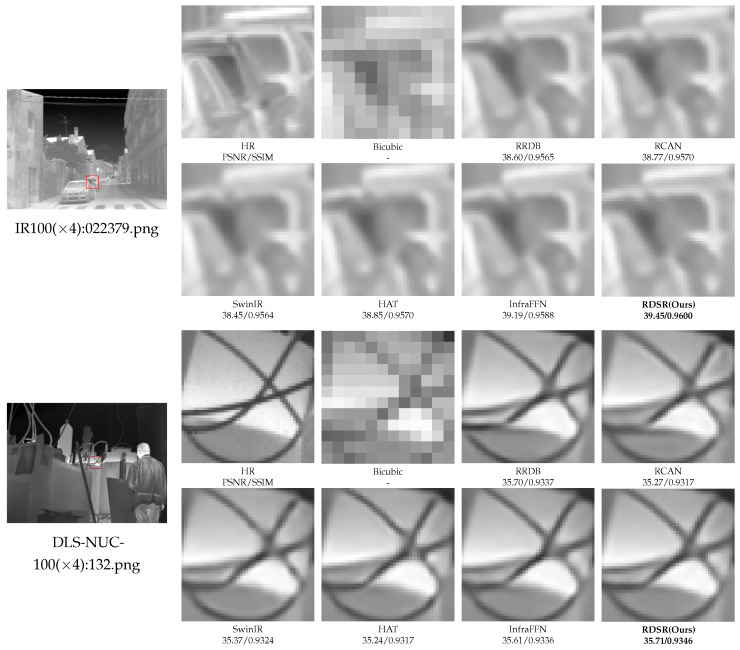
Visual comparison results of different models on different datasets under ×4 superresolution. The image patches used for comparison are marked with red boxes in the original images.

To intuitively verify the reconstruction performance of the proposed RDSR model in infrared image super-resolution tasks, this paper conducted a ×4 super-resolution qualitative comparison experiments on four typical infrared datasets: IR700, IR100, Flir, and DLS-NUC-100. These datasets represent a wide range of thermal imaging scenarios, from urban surveillance to industrial inspections, each presenting unique challenges such as low contrast and thermal noise. Representative image patches were selected, and their visual effects were compared with those of mainstream methods, such as Bicubic, RRDB [18], RCAN [7], SwinIR [8], HAT [10], and InfraFFN [11]. The results are shown in Figure 5.

Overall, the Bicubic exhibits obvious blurring and loses almost all high-frequency components. While CNN-based methods like RRDB and RCAN improve sharpness, they tend to cause over-smoothing and significant detail loss at structural edges due to the limited receptive field. Transformer-based models such as SwinIR and HAT can enhance structural continuity to a certain extent by leveraging long-range dependencies; however, they often suffer from texture adhesion or “grid artifacts” in high-frequency regions with weak thermal contrast. InfraFFN, specifically designed for infrared images, outperforms the above baseline methods in texture recovery, but its local structural consistency and edge sharpness remain weaker than those of the proposed RDSR.

The proposed method achieves clearer edges, more complete fine textures, and fewer artifacts across all representative scenarios. As evidenced by the quantitative values in Figure 5, RDSR reaches 33.84 dB/0.9207 on “698.png” (IR700), exhibiting superior structural similarity in regular periodic texture regions where other models fail to maintain line integrity. In “82.png”, our model depicts the contours of small targets and their surrounding thermal radiation structures more accurately, which is crucial for target recognition. For “0044.png” of the Flir dataset, the proposed method maintains the complex thin line structures of the fence more completely, showing more stable line directions and better width consistency. In “022379.png” of the IR100 dataset, the RDSR presents clearer vehicle body edges, contour structures, and more complete boundaries, effectively suppressing the “halo” effect common in thermal imaging. Finally, for “132.png” of the DLS-NUC-100 dataset, the model successfully recovers the intersecting thin line details with sharper boundaries and stronger continuity. In summary, the visual comparison in Figure 5 confirms that the RDSR effectively improves detail recovery and structural fidelity, demonstrating robust generalization across diverse infrared imaging conditions.

#### 4.3.3. Model Complexity

To evaluate the computational cost and performance gain of the proposed method in infrared image super-resolution tasks, Table 3 presents the comparison results of complexity tests for different methods on an NVIDIA GeForce RTX 4090D GPU. The test indicators include single inference running time (Running), number of parameters (Number of parameters), and Floating-Point Operations (FLOPs), with the PSNR/SSIM on IR700_test (×4) also provided for reference.

It can be seen from Table 3 that traditional convolution-based baseline models (SRResNet, EDSR) have a low number of parameters and low computational complexity, along with a fast inference speed, but their reconstruction accuracy significantly lags behind subsequent deeper networks. With the deepening of network structures and the introduction of more complex attention mechanisms (such as RRDB, RCAN, RDN, NLSN, HAN), the number of model parameters and FLOPs increase significantly, and the running time also rises accordingly. Among them, HAN and NLSN have large scales and high computational costs. Transformer-based methods (SwinIR, HAT) maintain a relatively controllable complexity level at high accuracy, but they still incur considerable inference time overhead overall.

In the above comparison, the proposed method RDSR achieves the best reconstruction accuracy under the premise of medium complexity, which is superior to existing comparison methods, and the inference time (0.0622 s) is kept within an acceptable range. Compared with the infrared-related method InfraFFN with high accuracy, the proposed method achieves higher reconstruction quality with similar parameter scale and computational complexity, and has a shorter running time. Compared with high-cost models (such as HAN, NLSN), the proposed method obtains better PSNR/SSIM with a lower number of parameters and FLOPs, reflecting a higher performance–complexity ratio. Although our model achieves good reconstruction accuracy in various indicators, its number of parameters and FLOPs are still at a medium to high level. This result indicates that the proposed method still has deficiencies in computational efficiency.

#### 4.3.4. Statistical Significance and Generalization Analysis

To further substantiate the reliability and superiority of the proposed RDSR, we conducted a rigorous statistical significance study. A paired t-test was performed on 50 representative samples from the IR700 test set at ×4 scale, comparing RDSR against the baseline InfraFFN and the state-of-the-art SwinIR. As summarized in the statistical analysis, the performance gains achieved by RDSR are highly significant. Specifically, the *p*-values for PSNR and SSIM compared to InfraFFN are $7.40\times 10^{-5}$ and $1.13\times 10^{-6}$, respectively. Even when compared with the competitive SwinIR, the *p*-values reach remarkably low levels ($p<10^{-13}$ for both me trics). These results, characterized by p<0.001, statistically demonstrate that the improvements in RDSR are consistent across diverse image contents and are not attributable to random fluctuations. Combined with the stable performance across multiple independent datasets (e.g., FLIR and DLS-NUC-100), this provides compelling evidence for the robust generalization and architectural effectiveness of our dual-branch interaction design.

### 4.4. Ablation Experiments

This work is improved upon in the InfraFFN framework, with key modifications to the spatial interaction module and the traditional MLP module in FFN. The proposed BDSI replaces the original spatial interaction module, and MSSA replaces the MLP module in the feed-forward network (FFN). Table 4 presents the ablation results under different module combinations to evaluate the impact of the proposed BDSI and MSSA modules. The results show that when only the BDSI module is used, the model has more parameters and higher computational complexity, but achieves improved performance on all five test sets. This indicates that the BDSI module can effectively improve reconstruction quality but incurs higher computational costs. When only the MSSA module is used, both the model complexity and the number of parameters are reduced, and the model performance can be maintained stably, demonstrating that the MSSA module can bring effective performance gains while reducing computational complexity. When both the BDSI and MSSA modules are used, the model achieves the optimal performance metrics on all five test sets while maintaining medium complexity. This indicates that the two modules are complementary, and their combined use can further improve the overall performance of the model and achieve a better balance between performance and complexity.

## 5. Conclusions

This work presents an infrared image super-resolution network RDSR integrating a CNN–Transformer hybrid architecture, aiming to enhance global modeling and local detail reconstruction performance. RDSR takes a residual dense network as the backbone and constructs a parallel dual-branch structure of convolution and self-attention. It strengthens the spatial information flow between branches through the designed BDSI dual-branch spatial interaction module; additionally, it proposes the MSSA multi-scale separable spatial aggregation module to achieve lightweight multi-scale feature enhancement. Extensive experiments have verified the effectiveness and reliability of RDSR. However, a limitation of RDSR is its relatively high number of parameters and computational complexity. Future research will focus on reducing the model parameter count and computational complexity, facilitating the edge deployment of the model on resource-constrained embedded infrared devices.

## Figures and Tables

**Table 1:** Quantitative comparison with existing super-resolution models under ×2 and ×4 scales on different test sets. Our model’s metrics are highlighted in bold [5,6,7,8,9,10,11,17,18,19,41,42].

Method	Scale	IR700_Test		Flir		IR100		Results-A		DLS-NUC-100	
		PSNR	SSIM	PSNR	SSIM	PSNR	SSIM	PSNR	SSIM	PSNR	SSIM
SRResNet	×2	39.51	0.9524	43.16	0.9860	43.83	0.9684	37.89	0.9351	40.29	0.9292
EDSR		39.57	0.9527	43.20	0.9861	43.84	0.9684	37.88	0.9350	40.29	0.9293
RRDB		39.73	0.9533	43.23	0.9862	43.84	0.9684	37.90	0.9352	40.31	0.9294
RDN		39.73	0.9539	43.26	0.9862	43.85	0.9684	37.89	0.9352	40.31	0.9294
RCAN		39.81	0.9536	43.23	0.9861	43.82	0.9683	37.88	0.9349	40.29	0.9292
HAN		39.82	0.9535	43.27	0.9862	43.86	0.9684	37.83	0.9348	40.32	0.9292
NLSN		39.72	0.9530	43.29	0.9863	43.87	0.9684	37.87	0.9350	40.37	0.9296
SwinIR		39.80	0.9532	43.33	0.9864	43.87	0.9684	37.91	0.9354	40.40	0.9296
HAT		39.85	0.9534	43.32	0.9864	43.86	0.9685	37.91	0.9353	40.40	0.9296
SRFormer		39.79	0.9532	43.27	0.9863	43.82	0.9684	37.89	0.9354	40.33	0.9292
DAT		39.80	0.9533	43.34	0.9863	43.86	0.9684	37.92	0.9354	40.39	0.9295
InfraFFN		40.06	0.9544	40.37	0.9865	43.88	0.9686	37.93	0.9355	40.41	0.9297
**RDSR (Ours)**		**39.87**	**0.9536**	**43.38**	**0.9865**	**43.91**	**0.9686**	**37.96**	**0.9357**	**40.43**	**0.9299**
SRResNet	×4	31.67	0.8551	35.03	0.9178	39.39	0.9406	33.23	0.8321	36.28	0.8831
EDSR		31.70	0.8559	35.03	0.9181	39.44	0.9410	33.22	0.8325	36.28	0.8833
RRDB		31.90	0.8586	35.20	0.9201	39.53	0.9415	33.28	0.8334	36.40	0.8842
RDN		31.90	0.8583	35.21	0.9201	39.59	0.9417	33.28	0.8333	36.38	0.8841
RCAN		31.93	0.8587	35.24	0.9205	39.60	0.9419	33.29	0.8336	36.38	0.8842
HAN		32.02	0.8600	35.16	0.9194	39.60	0.9417	33.27	0.8328	36.38	0.8838
NLSN		31.97	0.8597	35.17	0.9195	39.58	0.9418	33.28	0.8333	36.41	0.8842
SwinIR		32.04	0.8616	35.23	0.9207	39.59	0.9417	33.28	0.8337	36.44	0.8845
HAT		32.09	0.8630	35.32	0.9215	39.66	0.9421	33.33	0.8342	36.46	0.8847
SRFormer		32.07	0.8617	35.30	0.9212	39.67	0.9421	33.30	0.8345	36.49	0.8848
DAT		32.08	0.8615	35.34	0.9215	39.69	0.9423	33.35	0.8346	36.47	0.8849
InfraFFN		32.21	0.8637	35.41	0.9229	39.75	0.9426	33.36	0.8348	36.56	0.8857
**RDSR (Ours)**		**32.36**	**0.8651**	**35.54**	**0.9242**	**39.84**	**0.9431**	**33.42**	**0.8357**	**36.66**	**0.8864**

**Table 2:** Quantitative comparison with existing infrared image super-resolution models under ×2 and ×4 scales on different test sets. Our model’s metrics are highlighted in bold [11,21,36,38,43,44].

Method	Scale	IR700_Test		Flir		IR100		Results-A		DLS-NUC-100	
		PSNR	SSIM	PSNR	SSIM	PSNR	SSIM	PSNR	SSIM	PSNR	SSIM
CNNSC	×2	37.45	0.9413	42.13	0.9833	43.41	0.9674	37.50	0.9328	39.44	0.9254
DASR		39.74	0.9531	43.31	0.9863	43.84	0.9683	37.92	0.9353	40.39	0.9296
ChaSNet		39.63	0.9528	43.26	0.9862	43.49	0.9590	37.92	0.9355	40.34	0.9295
PSRGAN		35.31	0.9159	40.41	0.9730	41.81	0.9529	36.20	0.9114	38.14	0.9044
IRSRMamba		33.39	0.9004	35.33	0.9169	38.14	0.9321	33.01	0.8722	36.99	0.9021
InfraFFN		40.06	0.9544	43.37	0.9865	43.88	0.9686	37.93	0.9355	40.41	0.9297
**RDSR (Ours)**		**39.87**	**0.9536**	**43.38**	**0.9865**	**43.91**	**0.9686**	**37.96**	**0.9357**	**40.43**	**0.9299**
CNNSC	×4	30.09	0.8221	33.85	0.8996	38.19	0.9336	32.59	0.8177	35.01	0.8715
DASR		31.90	0.8603	35.21	0.9208	39.59	0.9417	33.23	0.8333	36.35	0.8842
ChaSNet		31.82	0.8580	35.12	0.9188	39.53	0.9415	33.30	0.8336	35.03	0.8381
PSRGAN		29.29	0.7887	32.89	0.8764	36.27	0.9001	31.80	0.8001	33.58	0.8378
IRSRMamba		29.65	0.8153	31.63	0.8498	35.35	0.9021	30.50	0.7897	33.68	0.8510
InfraFFN		32.21	0.8637	35.41	0.9229	39.75	0.9426	33.86	0.8348	36.56	0.8857
**RDSR (Ours)**		**32.36**	**0.8651**	**35.54**	**0.9242**	**39.84**	**0.9431**	**33.42**	**0.8357**	**36.66**	**0.8864**

**Table 3:** Comparison of model complexity with existing methods. We conducted complexity tests for super-resolution tasks on an NVIDIA GeForce RTX 4090D GPU, with test indicators including running time, number of parameters, and Floating-Point Operations (FLOPs) [5,6,7,8,9,10,11,17,18,19,21,41,43].

Method	Scale	Running Time (s)	Number of Parameters (M)	FLOPs (G)	IR700_Test	
					PSNR	SSIM
SRResNet	×4	0.0025	1.331	10.464	31.67	0.8551
EDSR		0.0015	1.811	9.251	31.70	0.8559
RRDB		0.0306	16.696	73.353	31.90	0.8586
RDN		0.0088	22.269	93.019	31.90	0.8583
RCAN		0.0410	15.590	65.171	31.93	0.8587
HAN		0.0556	64.194	268.367	32.02	0.8600
NLSN		0.0159	44.147	209.876	31.97	0.8597
SwinIR		0.0118	11.847	50.458	32.04	0.8616
HAT		0.0355	20.506	85.619	32.09	0.8618
DAT		0.0256	3.808	17.172	32.08	0.8615
DASR		0.0410	21.250	88.890	31.90	0.8603
ChaSNet		0.0238	14.477	59.053	31.82	0.8580
InfraFFN		0.0571	23.804	94.221	32.21	0.8637
**RDSR (Ours)**		**0.0622**	**23.658**	**96.75**	**32.36**	**0.8651**

**Table 4:** Ablation experiments for different modules. The best results are highlighted in bold.

BDSI	MSSA	Params (M)	FLOPs (G)	IR700_Test		Flir	IR100	Results-A	DLS-NUC-100
				PSNR	SSIM	PSNR/SSIM	PSNR/SSIM	PSNR/SSIM	PSNR/SSIM
×	×	23.804M	94.221G	32.21	0.8637	35.41/0.9242	39.75/0.9426	33.36/0.8348	36.56/0.8857
√	×	24.588M	100.621G	32.28	0.8645	35.51/0.9237	39.79/0.9429	33.40/0.8352	36.64/0.8862
×	√	**22.921M**	**93.808G**	32.31	0.8649	35.51/0.9238	39.83/0.9431	33.41/0.8356	36.65/0.8862
√	√	23.657M	96.752G	**32.36**	**0.8651**	**35.54/0.9242**	**39.84/0.9432**	**33.42/0.8357**	**36.66/0.8864**

## Data Availability

Dataset available on request from the authors.
